# Phase Separation-Mediated Chromatin Organization and Dynamics: From Imaging-Based Quantitative Characterizations to Functional Implications

**DOI:** 10.3390/ijms23148039

**Published:** 2022-07-21

**Authors:** Woei Shyuan Ng, Hendrik Sielaff, Ziqing Winston Zhao

**Affiliations:** 1 Department of Chemistry, Faculty of Science, National University of Singapore, Singapore 119543, Singapore; w.shyuan@u.nus.edu (W.S.N.); hesielaf@nus.edu.sg (H.S.); 2 Centre for BioImaging Sciences (CBIS), Faculty of Science, National University of Singapore, Singapore 117557, Singapore; 3 Mechanobiology Institute (MBI), National University of Singapore, Singapore 117411, Singapore

**Keywords:** phase separation, chromatin organization, nuclear condensate, intrinsically disordered region, transcription, DNA damage repair, super-enhancer, quantitative imaging

## Abstract

As an effective and versatile strategy to compartmentalize cellular components without the need for lipid membranes, phase separation has been found to underpin a wide range of intranuclear processes, particularly those involving chromatin. Many of the unique physico-chemical properties of chromatin-based phase condensates are harnessed by the cell to accomplish complex regulatory functions in a spatially and temporally controlled manner. Here, we survey key recent findings on the mechanistic roles of phase separation in regulating the organization and dynamics of chromatin-based molecular processes across length scales, packing states and intranuclear functions, with a particular emphasis on quantitative characterizations of these condensates enabled by advanced imaging-based approaches. By illuminating the complex interplay between chromatin and various chromatin-interacting molecular species mediated by phase separation, this review sheds light on an emerging multi-scale, multi-modal and multi-faceted landscape that hierarchically regulates the genome within the highly crowded and dynamic nuclear space. Moreover, deficiencies in existing studies also highlight the need for mechanism-specific criteria and multi-parametric approaches for the characterization of chromatin-based phase separation using complementary techniques and call for greater efforts to correlate the quantitative features of these condensates with their functional consequences in close-to-native cellular contexts.

## 1. Introduction

The cell nucleus is known to be a highly crowded environment in which a myriad of biochemical reactions take place simultaneously. Hence, compartmentalization of intranuclear components and processes is an essential and effective strategy to achieve precise spatio-temporal coordination of such complex dynamics. The nucleolus and Cajal bodies, which were discovered over a century ago [1,2,3], are among the most conspicuous and structurally stable membraneless compartments observed within the nucleus. The constituents of these compartments were later found to be highly dynamic rather than static protein aggregates [4,5,6], although the underlying physical nature of these compartments was not clearly understood. Since evidence of a liquid-like state was demonstrated for P granules in germ cells of *Caenorhabditis elegans* [7], a growing number of membraneless nuclear bodies/structures, including paraspeckles [8,9], nuclear speckles [10], promyelocytic leukemia (PML) bodies [11] and DNA damage repair foci [12], have been revisited through the lens of phase separation, which has greatly expanded and re-shaped our understanding of the importance of intranuclear compartmentalization. As a unifying conceptual framework accounting for the formation and unique physico-chemical properties of such membraneless compartments, phase separation has emerged as a general mechanism that underpins a wide range of intracellular processes both inside and outside of the nucleus and involves a variety of biomolecular species [13,14,15,16,17]. In particular, adding on to the many types of phase separation phenomena discovered earlier that involve RNAs and RNA-binding proteins [9,18,19,20,21,22,23,24,25,26] or are implicated in processes related to RNA metabolism [27,28,29], more recent studies have uncovered the involvement of phase separation in regulating DNA- or chromatin-based molecular transactions. Here, we survey key recent findings on this growing body of phase separation-mediated phenomena specifically related to chromatin-based intranuclear processes, as revealed primarily through various quantitative imaging methods, and illustrate the critical functional roles of phase separation in regulating the organization and dynamics of these processes. More importantly, by illuminating the complex interplay between chromatin and various chromatin-interacting molecular players mediated by phase separation, this review sheds important light on an emerging multi-scale, multi-modal and multi-faceted landscape that hierarchically organizes the eukaryotic genome within the highly crowded and dynamic nuclear space.

## 2. Intranuclear Phase Separation: Physico-Chemical Properties and Molecular Driving Forces

Just as oil tends to “demix” with water, chemically and structurally distinct biomolecules that exist as a homogenously mixed solution within the cell can similarly separate themselves into distinct and stably co-existing phases, each enriched with a distinct composition and/or concentration of biomolecules, resulting in liquid-like droplets known as biomolecular condensates (or simply condensates) [14]. The existence of the liquid–liquid phase separation (LLPS) of proteins was first observed and correlated to the physics of phase transition using lysozyme as the model system [30], which subsequently paved the way for understanding LLPS as the physico-chemical underpinning of certain pathological states (e.g., cataracts) [31,32]. However, despite the demonstration of its disease implications, it was only until recently that LLPS re-emerged as a new framework for conceptualizing membraneless intracellular organelles, hence encouraging biologists to revisit many of its previously under-explored properties.

The formation of biomolecular condensates can be best understood from the perspective of the thermodynamics and kinetics underlying polymer demixing in solution, a concept firmly rooted in soft matter physics. Put simply, biomolecules can be driven to phase separate by the balance between two counteracting thermodynamic properties: entropy (which favors the well-mixed state) and enthalpy (in the form of attractive interactions between them). Beyond a particular concentration threshold, at which point interactions between the biomolecules exceed their interactions with the solvent (i.e., the cytoplasm or nucleoplasm of a cell) as a consequence of molecular enrichment, the biomolecules become less and less soluble and thus separate into phases with different concentrations but the same chemical potential to minimize the overall free energy of the system. At the same time, perturbations such as alterations in biomolecular structure or affinity and environmental changes that shift the equilibrium of the system can lead to changes in the material and/or physico-chemical properties of the condensates. Such behaviors have key functional consequences in various biological contexts, where condensates enriched with certain biomolecular species can assemble at specific intracellular locations to perform specialized tasks and readily disassemble in a regulated manner.

**Figure 1:**
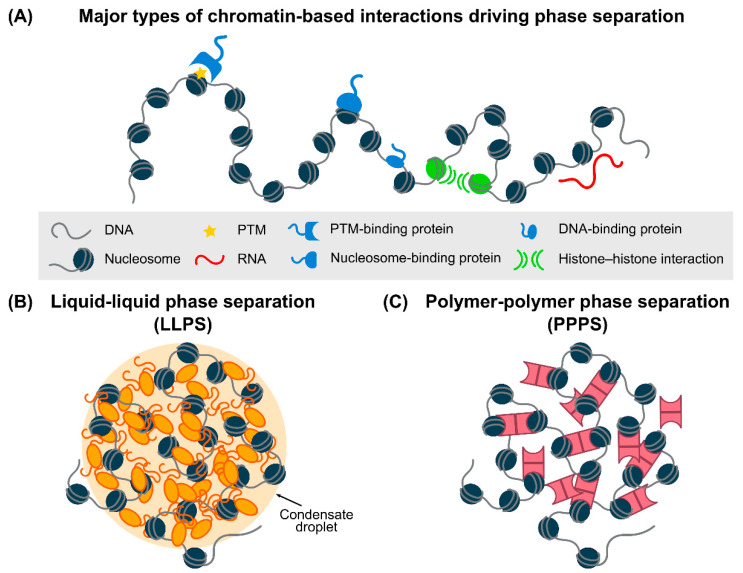
Schematic representation of chromatin-based phase separation. (**A**) Major types of chromatin–protein and chromatin–RNA interactions that can drive chromatin-based phase separation, including direct binding of proteins or RNAs to DNA/nucleosomes or to post-translational modifications (PTMs), as well as inter-nucleosome or -histone tail interactions. (**B**) Liquid–liquid phase separation as promoted by weak and multivalent interactions between chromatin and chromatin-associated factors (orange). (**C**) Polymer–polymer phase separation takes place through the oligomerization of multiple bridging proteins (pink) that draw different regions of the chromatin scaffold together via nonspecific interactions.

While biomolecular condensates are diverse in their molecular make-ups, intracellular locations and functions, they often share a similar set of physico-chemical properties in terms of morphology, dynamics and assembly/disassembly behaviors. To begin with, phase condensates often exhibit the characteristics of liquid-like droplets (e.g., spherical in shape, tendency to coalesce and low surface tension) and can exist stably while being able to dynamically alter their compositions in response to environmental conditions via molecular exchange with the surrounding cellular milieu [33,34]. Secondly, molecular enrichment within such condensates is often driven by preferential interactions between proteins, RNAs and DNA (Figure 1A), particularly multivalent interactions that can be achieved via repetitive modules [14,35]. These modules harbor multiple elements for intra- or inter-molecular interactions, in line with the classic polymer physics descriptions of multivalent molecules in a mixture. Associated with multivalency is a molecular feature known as an intrinsically disordered region (IDR), a type of protein domain with low structural complexity that is often enriched with specific amino acid residues, repetitive motifs or patches of alternating charges. IDRs are commonly implicated in LLPS, in which the formation and selective partitioning of condensates is attributed to transient and weak interactions between IDR-containing biomolecules, including π–π stacking, π–cation interaction, Van der Waals forces, hydrogen bonding and electrostatic and hydrophobic interactions [36]. Modular proteins can also act as scaffolds when recruiting clients that harbor IDRs, which in turn form a multi-modal interaction network to enhance the avidity of weak interactions in the condensed phase [37,38]. In addition to IDRs, oligomerization domains have also recently been shown to enhance the LLPS of protein domains and can potentially serve as an alternative molecular signature associated with LLPS [39].

In the context of chromatin (Figure 1B), LLPS can drive the formation of chromatin-associated liquid-like droplets via electrostatic attractions between charged residues, dipoles or aromatic groups. In addition to multivalency, site-specific phase condensation can also be promoted and tuned using DNA, RNA and free nucleotides [19,40,41,42]. In particular, repetitive DNA sequences and the epigenetic states of chromatin can modulate the nucleation and dynamics of intranuclear condensates, contributing to chromatin compaction and other chromatin-based processes. For instance, CpG islands (i.e., CG-rich DNA sequence elements) can recruit the Polycomb repressive complex 2 (PRC2) for the maintenance of the stability of repressed genome at these sites [43], which has been subsequently found to be involved in phase separation (see below for details). LLPS of repetitive telomeric DNA sequences is also implicated in the induction of alternative lengthening of telomeres (ALT) [44], as well as in promoting ALT-dependent telomere maintenance [45].

Alternatively, polymer–polymer phase separation (PPPS), also known as bridging-induced phase separation, can take place through the oligomerization of multiple modular or bridging proteins that link different regions of the chromatin scaffold together via nonspecific interactions (Figure 1C). The molecular compositions inside and outside of the condensate formed by PPPS are the same and do not impact the size of the condensate formed, as opposed to LLPS in which changes in the concentration of multivalent binders can affect the size of the condensate. In addition, LLPS droplets have been predicted to be able to persist after the removal of chromatin scaffolds, whereas PPPS condensates rely on chromatin scaffolds for their formation [46]. PPPS was first conceived theoretically using polymer physics models and demonstrated via simulations. For example, in the “strings and binders switch” model, diffusible binding factors establish interactions between binding sites on nonrandom chromatin conformations, leading to stable chromatin architectures [47,48]. On the other hand, PPPS can also be driven by entropic bridging-induced attractions through local DNA distortions induced by bridging proteins that bridge distant DNA regions together; the associated entropic penalties can be minimized by clustering these distorted elements, which results in a local increase in DNA concentration to attract more bridging molecules into the condensate [49,50,51]. Recently, PPPS has been shown to underlie the formation of DNA–cohesin clusters in vivo [52], pointing to the potential applicability of this previously under-explored mechanism of phase separation in various DNA–protein complexes.

Since the theoretical framework [13,53,54,55] and the various computational models [56] for understanding chromatin-based phase separation have been expertly reviewed elsewhere, we focus here instead on their quantitative characterizations via imaging-based approaches, as well as their functional implications in organizing and regulating intranuclear structures and processes. Even though the physical processes that underlie LLPS and PPPS can be separated well in theory and simulations, distinguishing between them is often hampered in practice by experimental limitations, and most of the studies reviewed here do not make a specific distinction between these two mechanisms.

## 3. Quantitative Imaging Techniques for Probing Chromatin-Based Phase Condensates

Over the years, a variety of technical approaches have been employed to characterize chromatin-based phase separation from different fronts, including in vitro biochemical reconstitution, optical imaging (both in cellulo and in vivo) and genomic methodologies (e.g., Hi-C, ChIP-seq and ATAC-seq), as well as theoretical/computational modeling. Among these, optical imaging-based approaches (in both fixed and live samples) arguably provide the most direct and comprehensive capabilities for the in situ quantification of these phase condensates across a wide range of spatial and temporal scales, as has been demonstrated for other intranuclear processes [57]. Despite their respective capabilities, advantages and limitations (Table 1), most of these techniques rely on the use of fluorescent proteins or dyes (via, e.g., SNAP, CLIP and Halo-tags [58,59,60]) for the labeling and visualization of condensate components inside the cell. In addition to the more conventional imaging configurations (such as wide-field and confocal), many of these techniques also employ total internal reflection fluorescence (TIRF) or light-sheet illuminations in order to leverage their superior optical sectioning capabilities and therefore achieve enhanced sensitivity.

**Figure 2:**
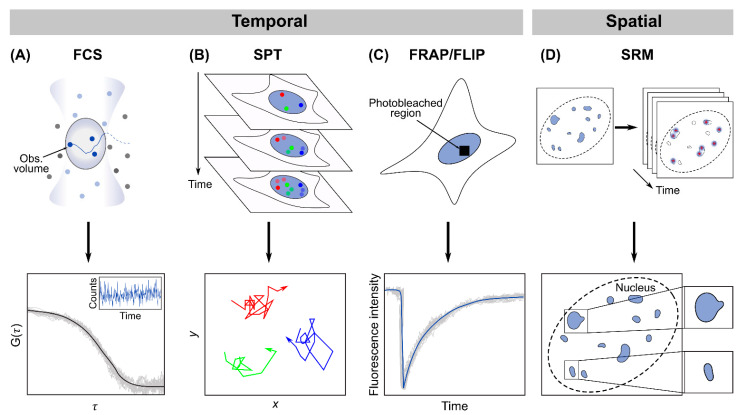
Principles of major types of quantitative imaging techniques commonly used for characterizing chromatin-based phase condensates. (**A**) FCS quantifies intranuclear dynamics by monitoring the fluorescence intensity fluctuations as biomolecules move in and out of a small observation volume; a typical intensity trace (inset) and the autocorrelation function curve calculated from it are shown. (**B**) SPT detects individual fluorescent biomolecules inside the nucleus and tracks their movements over time; a few typical single-particle trajectories (colored red, green and blue) are depicted. (**C**) Photobleaching-based techniques, such as FRAP and FLIP, where a small region of the cell nucleus is selectively photobleached; a typical FRAP curve is shown. (**D**) The SRM technique PALM/STORM labels an intranuclear structure with photoswitchable fluorophores, activates a random subset of the fluorophores each time and localizes their individual positions with ultra-high spatial precision; iterating the process multiple times then reconstructs a super-resolution image of the structure.

In the time domain, a powerful technique for quantifying the dynamics of chromatin-based phase condensates is fluorescence correlation spectroscopy (FCS), which monitors the fluctuations in fluorescence intensity produced by molecules as they diffuse across a small confocal observation volume, followed by autocorrelation analysis of these time traces and model fitting to extract quantitative parameters (Figure 2A) [61,62]. Combining FCS with photoactivatable fluorescent proteins (paFCS) enables us to fine-tune the level of fluorescent molecules detected, hence making it suitable for probing high-background intracellular environments, such as the nucleus [63]. Other related fluctuation-based techniques include polarization-sensitive FCS [64], number and brightness (N&B) analysis [65,66] and imaging FCS and raster image correlation spectroscopy (RICS) [67,68], each of which is suitable for quantifying a particular aspect of condensate dynamics. A complementary technique to FCS is single-particle tracking (SPT), which leverages the ability to detect the fluorescence signal of individual biomolecules to precisely localize their positions and track their dynamics over time (Figure 2B) [69]. The sensitivity of SPT, especially when measuring inside the highly crowded cell nucleus, can be enhanced through integration with various light-sheet-based illumination schemes [70,71,72,73], which selectively excite only a thin section of the nucleus to cut down the out-of-focus background that could easily overwhelm the signal of a single biomolecule. Finally, photobleaching-based techniques, such as fluorescence recovery after photobleaching (FRAP) and fluorescence loss in photobleaching (FLIP), probe intranuclear dynamics by photobleaching the fluorescent molecules in a specific region of the nucleus and then monitoring either the recovery of fluorescence as bleached molecules in the region get replenished after a single photobleaching (FRAP) (Figure 2C) or the propagation of fluorescence loss through the nucleus after repeated photobleaching (FLIP) [74,75].

In the spatial realm, super-resolution microscopy (SRM) has been widely used to characterize the spatial features of chromatin-based phase condensates at resolutions an order of magnitude below those afforded by conventional imaging techniques (such as confocal microscopy). Among the various approaches for breaking the diffraction limit, single-molecule localization-based methods, such as photoactivated localization microscopy (PALM) and stochastic optical reconstruction microscopy (STORM), leverage the labeling of a cellular structure with photoswitchable or photoactivable fluorophores, a sparse subset of which can be randomly activated, individually resolved and localized with nanometer precision. Iterating the process multiple times with a different subset of fluorophores activated each time allows a super-resolution image to be reconstructed from the collective localizations of all fluorophore molecules in the target structure (Figure 2D) [76,77,78]. Alternatively, methods based on spatially patterned illumination, such as structured illumination microscopy (SIM) and stimulated emission depletion (STED) microscopy, make use of sub-diffraction-limit spatial features introduced into the excitation light either to generate Moiré patterns from cellular structures that can be used to reconstruct a super-resolution image (SIM) [79] or to suppress fluorescence emission from fluorophores located off the center of the excitation region and effectively shrink the point spread function (STED) [80]. Similar to SPT, these super-resolution techniques can also be combined with various implementations of light-sheet illumination [73,81,82,83], especially when resolving highly dense intranuclear structures, such as those involving chromatin. In addition, DNA or RNA fluorescence in situ hybridization (FISH) enables us to spatially correlate chromatin-based phase condensates with their genomic locations or transcriptional outputs, although no dynamic information can be obtained due to the need for cell fixation.

**Table 1:** Commonly used quantitative imaging techniques for the characterization of chromatin-based phase condensates and their respective capabilities, advantages and limitations.

Technique	Condensate Parameters Measurable	Spatial/Temporal Resolutions		Sample Types Compatible	Pros and Cons	
FCS (and associated variants)	Diffusion coefficient Concentration Residence time for binding (e.g., to DNA) Local viscosity (polarization-sensitive FCS) Oligomerization state (N&B analysis) Spatial context of condensate dynamics (RICS and imaging FCS)	*Spatial:*	Diffraction-limited	Live cells/organisms (e.g., embryos)	*Pros:*	Wide coverage of temporal dynamics (from microseconds to seconds) Low photodamage/photobleaching to/of live samples due to low illumination power used
		*Temporal*:	Microseconds		*Cons:*	Poor signal quality could result from high molecular concentrations commonly found in condensates Difficult to probe condensates smaller than diffraction limit
SPT	Diffusion coefficient Residence time for binding (e.g., to DNA) Spatial context of condensate dynamics	*Spatial*:	Diffraction-limited (with nm localization precision)	Live cells/organisms (e.g., embryos)	*Pros:*	Direction visualization of condensate dynamics Less reliant on calibrations/corrections commonly required for other techniques
		*Temporal*:	Milliseconds		*Cons:*	Tracking duration can be limited by photobleaching (especially when using fluorescent proteins) Signal quality for single molecules can be reduced in high-background/dense intracellular environments
FRAP/ FLIP	Mobility (as measured by characteristic half-time for fluorescence recovery or loss) Local viscosity (indirectly derived)	*Spatial:*	Diffraction-limited	Live cells/organisms (e.g., embryos)	*Pros:*	More suitable for probing dynamics at longer timescales (seconds to minutes or longer)
		*Temporal:*	Seconds		*Cons:*	Requires complex data analysis/modeling Not suitable for probing fast and transient dynamics Ensemble nature masks intrinsic heterogeneities among individual biomolecules
SRM (e.g., PALM/STORM, SIM, STED)	Spatial/morphological features (e.g., size, area, aspect ratio); Intranuclear distribution and density Molecular stoichiometry Colocalization between components	*Spatial*:	10 s of nm or better	Fixed or live cells/ tissues	*Pros:*	Superior spatial resolution Possible to perform molecular counting
		*Temporal*:	Up to seconds (for live samples)		*Cons:*	Limited imaging speed/temporal resolution due to the need to accumulate sufficient localizations (PALM/STORM) Computationally demanding image reconstruction (SIM) Requires complex optical instrumentation and high laser power to achieve fluorescence depletion (STED)

While these imaging techniques are by no means specific to probing only intranuclear phase condensates (as opposed to condensates at other intracellular locations), they are nevertheless among the most widely adopted methods in previous studies of chromatin-based phase separation and are often used in combination with each other or in conjunction with other complementary approaches (such as in vitro biochemistry measurements of the concentration range associated with phase separation for a particular condensate). The extensive application of these quantitative methods has not only shed light on previously hidden physico-chemical parameters of a variety of chromatin-based phase condensates (as summarized in Table 2), but also in many cases led to illuminating insights into the mechanistic and functional implications of these phenomena, as will be discussed in detail in the following section.

## 4. Multi-Scale Chromatin Organization and Dynamics Mediated by Phase Separation

The eukaryotic genome is organized hierarchically in the nucleus across multiple length scales both in physical and sequence spaces [112,113]. At the finest scale (up to several kbs), DNA is first compacted into nucleosomes consisting of 147 bps of DNA wrapping around a histone octamer core. Nucleosomes are packed into 10 nm fibers and then organized into chromatin loop structures and topologically associating domains (TADs), which span several kbs to several Mbs [114,115]. Finally, chromatin domains assemble into A/B compartments (approximately corresponding to euchromatin and heterochromatin, respectively), which make up chromosomes that each occupy a distinct territory within the nuclear space, several µm in size and spanning hundreds to thousands of Mbs. Overall, phase separation has been implicated in the organization and dynamics of chromatin at each of these scales, which is consistent with its intrinsic propensity for phase separation as evidenced by the fact that nucleosome arrays are capable of condensing into liquid droplets in vitro under physiological conditions [41].

### 4.1. Large-Scale Chromatin Organization

In order to be packed into a certain nuclear domain or territory, chromatin needs to be organized into higher-order architectures, and the principles governing the process have been well-illustrated through theoretical modeling and computational simulations [116,117]. Starting from simple models that assume the chromatin fiber to be a self-avoiding polymer bead chain to account for the scaling properties of chromatin folding through binder-mediated interactions [47,118], the various models that have been developed to date are now capable of recapitulating the dynamic behaviors and properties of chromatin folding and its resulting architectural features, in good agreement with data from FISH and chromosome conformation capture experiments across different species [119,120,121]. Importantly, there is increasing in silico evidence supporting the role of phase separation in orchestrating genome compartmentalization by taking into account different types of biochemical interactions within a chromosome, where chromatin of the same epigenetic type tends to colocalize and adopt certain architectural conformations as a consequence of energetic stabilization [120,121,122,123]. These studies, coupled with the various experimental findings detailed below, are merging towards a unifying conception of phase separation as a key driving force of 3D genome organization, which has also been shown to be evolutionarily conserved across the three kingdoms of life [124].

**Figure 3:**
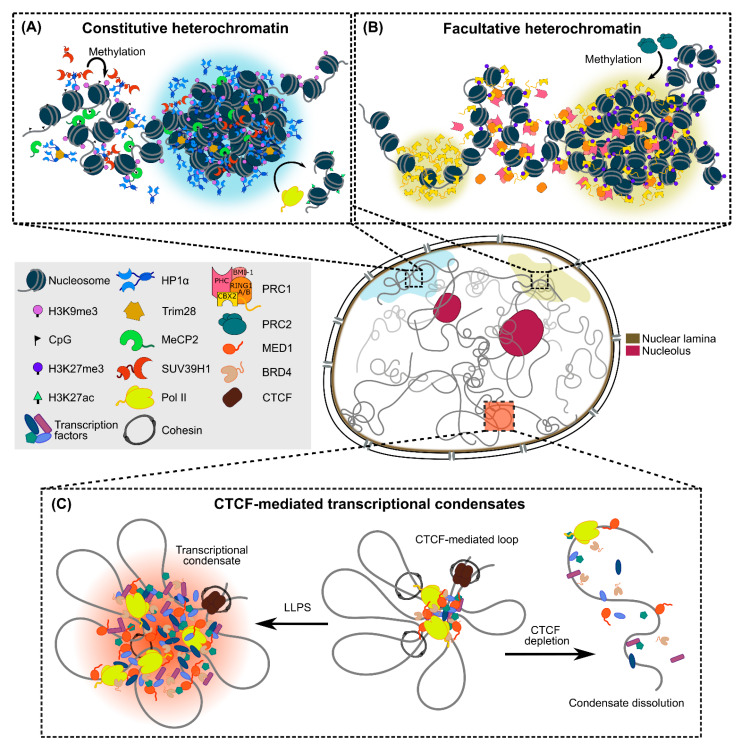
Intranuclear organization of chromatin via phase separation into large- and intermediate-scale condensates. (**A**) HP1α dimer binding to H3K9me3 on heterochromatin initiates condensate formation; further interactions with SUV39H1 and TRIM28 lead to higher-order oligomers that enhance LLPS to establish highly compact constitutive heterochromatin. Moreover, MeCP2 can also phase separate with HP1α, while the exclusion of H3K27ac and Pol II from the condensates further segregates the heterochromatin phase from the surrounding transcriptionally active regions. (**B**) Upon deposition of H3K27me3 marks on chromatin by PRC2, CBX2 binds to H3K27me3 and undergoes LLPS to establish facultative heterochromatin. The assembly of other PRC1 subunits further enhances the initial condensed phase into larger condensates. (**C**) CTCF-mediated chromatin looping provides an architectural framework for the local enrichment of various transcriptional machineries (e.g., Pol II, MED1 and BRD4) and drives the formation of transcriptional condensates via LLPS. Depletion of CTCF dissolves these condensates.

Among the different packing states of chromatin, phase separation of the densely packed and largely transcriptionally inactive heterochromatin can be driven by heterochromatin protein 1 (HP1) and chromobox homolog 2 (CBX2), which recognize histone marks H3K9me3 and H3K27me3, respectively (Figure 3A,B) [40,84,85,90]. In particular, heterochromatin can be further categorized into constitutive and facultative heterochromatin, with the former being more densely packed and containing few genes but relatively large amounts of tandem repeats, while the latter contains genes that are often found in a transcriptionally repressed state in the absence of specific developmental cues [125,126]. The discovery of the role of HP1 in driving LLPS of constitutive heterochromatin (marked by H3K9me3) in Drosophila melanogaster and mammalian cells has expanded the conventional assembly mechanism for heterochromatin domains beyond chromatin compaction [84], although contrary evidence exists that suggests that heterochromatin foci can also form independently of HP1-driven LLPS [64]. Further supporting its role in the phase separation-driven formation of heterochromatin, HP1 binding has been found to increase the accessibility and dynamics of embedded histone residues within the nucleosome for more multivalent interaction sites [127], thereby promoting the bridging of multiple nucleosomes together through HP1 oligomerization and enhancing inter-nucleosome interactions. Heterochromatin condensate formation can also be further enhanced by linker histone H1 and post-translational modifications of HP1. H1, whose condensates colocalize with HP1α in vivo [40], has been shown to compartmentalize nucleosomes and reduce their dynamics within the condensate [41], while the phosphorylated N-terminal domain (NTD) of human HP1α possesses an enhanced propensity for driving LLPS through formation of higher-order oligomers that are more effective in bridging nucleosomes together [85]. In addition, the number of available chromodomains (CDs) that interact with H3K9me3-marked nucleosomes has also been found to serve as another driver for phase separation-mediated heterochromatin formation, in which synergetic interactions between HP1α/β and other heterochromatin-related proteins (e.g., TRIM28 and SUV39H1) in a complex lead to enhanced multivalent CD–H3K9me3 interactions that can drive heterochromatin condensation [86]. While these findings were mainly demonstrated in vitro with relatively short nucleosome arrays, the inter- and intra-molecular multivalent interactions between chromatin and its associated proteins, as well as the coalescence of heterochromatin condensates, could potentially drive the propagation of heterochromatin domains observed in live cells [128], beyond the conventional mechanism of protein–protein binding/oligomerization.

Moreover, the intrinsic selectivity afforded by the combinations of macromolecular interactions through phase separation serves as a higher-level regulatory mechanism across different types of heterochromatin condensates. For instance, methyl-CpG-binding protein (MeCP2) condensates selectively incorporate HP1α and compete with H1 to form mutually exclusive and distinct heterochromatin foci [87,88]. DNA methylation (especially at CpG sites) is also a common feature of constitutive heterochromatin besides H3K9me3 marks and is known to negatively regulate transcription [129]. In this context (Figure 3A), the transcriptionally repressive effect could be attributed to phase separation of MeCP2, which binds strongly to highly methylated heterochromatic chromocenters and forms condensates via LLPS that are capable of excluding transcriptional machineries [87,88]. In addition, phase separation also kicks in when it comes to the overall maintenance of heterochromatin stability. Notably, condensates of the well-known DNA damage response (DDR) factor 53BP1 play an unexpected role in protecting heterochromatin from DNA damage in a HP1α-dependent manner [89].

Similarly, phase separation has also been implicated in the formation of facultative heterochromatin (marked by H3K27me3). The Polycomb repressive complex 1 (PRC1), which recognizes H3K27me3 marks, can mediate phase separation via its CBX2 and PHC subunits (Figure 3B), with the phosphorylation of CBX2’s IDR domain and oligomerization of PHC’s sterile alpha motif (SAM) being critical for driving the condensation process [90,91,130]. Intriguingly, CBX2 does not depend on H3K27me3 for phase separation, but rather nucleates on chromatin directly to assemble CBX2-PRC1 condensates to speed up the target search process of CBX2, thereby increasing its genomic occupancy to recruit more clients [131]. In fact, the chromatin compaction functionality of PRC1 is facilitated by CBX2, while other CBX proteins in PRC1 act as bridging factors that recognize and recruit H3K27me3-marked chromatin into CBX-PRC1 condensates [90,132]. In addition, PRC1 condensates nucleated at H3K27me3 have also been shown to drive ubiquitination of histone H2 for de novo recruitment of PRC2 [133], leading to the propagation of H3K27me3 marks that in turn recruit more PRC1 into the condensates and establish Polycomb domains in facultative heterochromatin via a positive feedback loop [90]. These distinct phase separation-based cofactor recruitment mechanisms and “scaffold–client” interactions function both independently and in synergy to establish dynamic and multifunctional heterochromatin domains. The collective effect of this complex interaction network might explain the observations from previous studies that heterochromatin droplets in vitro and in vivo often exhibit incomplete FRAP recovery and long recovery half-time [84,86,88,91], as well as incomplete dispersion upon 1,6-hexanediol treatment [84,89,90], properties that suggest that they are not purely liquid-like structures as predicted by the LLPS model.

In contrast to heterochromatin, the role of phase separation in driving the formation of euchromatin as an intranuclear compartment has been studied less extensively. However, it is known that heterochromatin and active transcriptional condensates (which are mostly found in euchromatic regions) often form distinct phases from each other. Moreover, interactions between heterochromatin, but not between euchromatin, have been found to drive the compartmentalization of whole cell nucleus [134]. In line with the fact that acetylation generally enhances chromatin, allowing it to adopt an “open” euchromatic configuration for higher genomic accessibility [135], H3K27-acetylated chromatin only phase separates in the presence of multi-bromodomain proteins, such as BRD4, and is immiscible with H3K27me3 droplets [41]. In addition, given that many transcriptional regulators and RNA-binding proteins harbor high levels of IDRs and have a high propensity to phase separate in euchromatic regions [14], phase separation in euchromatin generally occurs at smaller length scales and is largely associated with transcription-related condensates, as discussed in the following sections.

### 4.2. Intermediate-Scale Chromatin Organization

Going further down the length scale, the organization of chromatin into smaller self-interacting TADs has been conventionally understood from a CTCF- and cohesin-mediated DNA loop extrusion mechanism [136,137]. However, TADs have been recently suggested to be far more dynamic than previously thought, as CTCF and cohesin form transient protein complexes with varying chromatin-binding dynamics to facilitate the formation and dissolution of chromatin loops throughout the cell cycle [138]. Notably, CTCF has been shown to be able to self-associate in an RNA-mediated manner via its RNA-binding region for chromatin loop formation [139], which also mediates CTCF clustering to speed up its nuclear target search by forming ~200 nm-sized “transiently trapping zones” [140]. Similar genomic reorganization is also observed in cells entering senescence, where such clusters are grouped into large senescence-induced CTCF clusters for chromatin loop reshuffling [141]. While the exact physico-chemical mechanism underlying CTCF clustering in these cases warrants further investigation, a recent study has shown that rather than undergoing phase separation itself, CTCF clusters can drive the local spatial confinement of chromatin and serve as a structural framework or nucleation site to facilitate the assembly of LLPS-mediated transcriptional condensates (Figure 3C) [92]. Furthermore, members of the SMC protein family, such as cohesion, can also induce the phase separation of DNA–cohesin clusters (~1 µm in size) in yeast cells through bridging of long DNA segments at least 3 kb in length [52]. This is the first experimental demonstration of PPPS in a biological system in vivo and suggests a potentially new mechanism for chromatin loop stabilization at transient CTCF-bound sites.

### 4.3. Small-Scale Chromatin Organization

**Figure 4:**
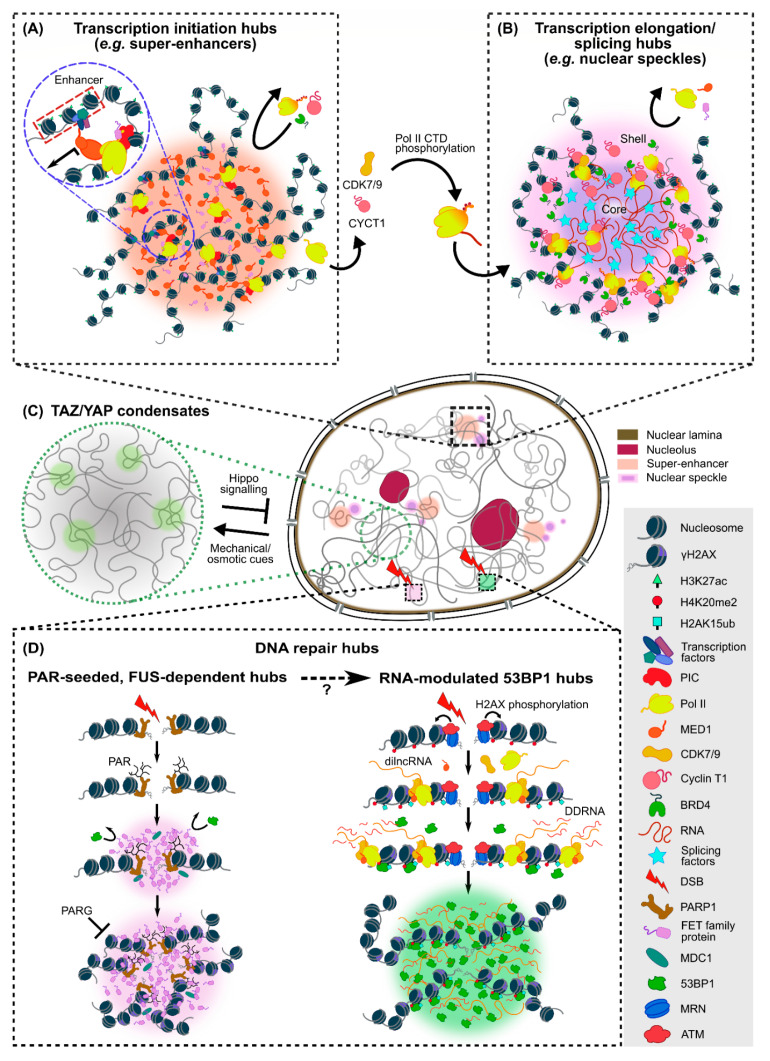
Phase separation-mediated small-scale chromatin-based condensates. (**A**) SE condensates serve as transcription initiation hubs that recruit TFs and coactivators (e.g., OCT4, c-MYC, KLF4 and MED1), which in turn recruit downstream transcriptional machineries. In particular, Pol II has a strong tendency to phase separate, but is excluded from these hubs upon phosphorylation of its CTD by CDK7/9. (**B**) Transcription elongation/splicing hubs formed by phosphorylated Pol II CTD, BRD4, transcription elongation factors (e.g., cyclin T1) and splicing factors, which are also found in nuclear speckles consisting of a core formed by long RNA transcripts and a shell decorated by chromatin and associated transcriptional elongation machineries. (**C**) Mechanical stress triggers LLPS of TAZ to initiate the transcription of TAZ-specific genes, while osmotic stress can induce the redistribution of YAP into the nucleus and reorganize chromatin to form YAP condensates for downstream gene transcription. TAZ/YAP condensates behave similarly to the SEs shown in (**A**) and can incorporate the transcriptional machineries for effective gene activation and transcription. (**D**) FET family proteins nucleate to drive LLPS of PAR-induced DNA repair hubs (left) during early DDR. FUS is required for the recruitment of DDR factors (such as 53BP1) to DNA damage sites and reorganizes phosphorylated histone variant γH2AX nano-foci into higher-order clusters, which can be dissociated by PAR glycohydrolase (PARG). In RNA-modulated 53BP1 repair hubs (right), DSB recognition by MRN initiates DDR response by recruiting ATM protein to phosphorylate H2AX. In addition, dilncRNA synthesized by Pol II at DSB sites can be further processed into small DNA damage response RNAs (DDRNAs), which support the nucleation of DDR foci by promoting LLPS of DDR factors into 53BP1-phase separated repair hubs. The relationship between FUS-dependent repair hubs and 53BP1 repair hubs is, however, not fully understood.

At the local level, phase condensates can nucleate at regions with either low or high chromatin density and selectively compartmentalize their interacting partners in close proximity to regulate chromatin-based processes, particularly transcription. Super-enhancers (SEs), which consist of a large number of enhancer elements drawn from distinct genomic regions into close proximity, are one of the earliest observed examples of such transcriptional condensates (Figure 4A). Many of the enhancer-associated factors including transcription factors (TFs), coactivators and chromatin regulators that correlate with SEs, such as BRD4, OCT4, FUS and MED1 (a subunit of the Mediator complex), are IDR-rich and capable of driving phase separation at SEs to activate gene transcription [97,98,106,108]. Specificity of gene expression can be achieved through selective interactions between the various TF condensates. Hence, phase separation of transcription-related proteins not only impacts chromatin organization by drawing enhancer elements together within the condensates [108] but can also lead to synchronous transcriptional bursting of multiple genes controlled by a shared enhancer [142]. Importantly, in order to differentially regulate transcriptional outputs in a precise manner, each phase separation-mediated system has its own optimal stoichiometric window for the most productive gene expression, depending on the type, level and strength of IDR–IDR interactions; perturbing such a balance could lead to aberrant or repressed transcription of target genes [102,103,143]. As such, phase separation observed at SEs or target gene loci at endogenous protein levels likely occurs at a more local scale, with condensates in the order of ~100 s nm in size (see Table 2), and couples specific TF interactions to local chromatin organization. On the one hand, the mechanical stiffness of local chromatin networks has been shown to affect the growth of transcriptional condensates, and serve as selective chromatin filters that lead to genomic rearrangements [144]. On the other hand, specific TF–DNA interactions can also initiate and stabilize condensates by organizing chromatin interactions at SE loci, as exemplified by the FET (FUS/EWS/TAF15) family protein EWS, which can form transactivation hubs that target GGAA microsatellites for aberrant oncogene activation and expression associated with Ewing’s sarcoma [104,145]. Furthermore, the key reprogramming factor KLF4, which recognizes specific promoter sequences, is able to mediate phase separation by bridging multiple DNA duplexes together, which in turn recruits other TFs and stabilizes long-range contacts of pluripotency-related genomic elements [106].

In addition to TFs, various components of the transcriptional machineries can also undergo phase separation to modulate the compartmentalization of chromatin and its interaction partners in the nuclear space. Firstly, RNA polymerase II (Pol II) and MED1 can both form small and transient (~100 nm in size and ~12 s in lifetime), as well as large and stable (>300 nm in size and >100 s in lifetime), clusters. The chromatin-associated stable clusters exhibit properties of phase condensates in which Pol II and MED1 colocalize at SEs that activate gene transcription [99], in line with earlier observations of the dynamic assembly of Pol II into heterogeneous populations of clusters that can correlate with transcription [83,146,147]. Moreover, the C-terminal domain (CTD) of Pol II can form condensates in both unphosphorylated and phosphorylated states that correspond to transcription initiation and elongation, respectively [42,111,148], and the phosphorylation status of Pol II CTD alters its selective partitioning into condensates for different transcriptional activities. Nascent Pol II CTD promotes the formation of TAF15 condensates by lowering the energetic barrier for its nucleation, which in turn recruits more Pol II into these transcription initiation hubs. In contrast, elongating Pol II CTD phosphorylated at Ser5 and Ser2 positions is excluded from TAF15 condensates but accumulates in concentrically adjacent regions [103]. These findings are in line with the earlier observation that phosphorylation dissolves Pol II CTD condensates, and the phosphorylated Pol II CTD is evicted from MED1 condensates [149]. Unphosphorylated Pol II CTD is also incorporated into MED1 condensates at SEs, while phosphorylation of Pol II CTD by CDK7/9 can drive its transition from the transcription initiation hubs to transcription elongation/splicing hubs [111]. Moreover, phosphorylated Pol II CTD is also recruited into cyclin T1 (a key component of nuclear speckles) condensates for enhanced phosphorylation of Pol II CTD and efficient transcription elongation [109,110] (Figure 4B). Taken together, these findings paint a general picture in which LLPS-mediated TF condensates draw a large number of enhancer elements together to stabilize the condensed phase, while mediating the formation of transcription initiation hubs by recruiting unphosphorylated Pol II CTD in the presence of short RNA transcripts produced from initial transcription. Upon phosphorylation, Pol II transitions to transcription elongation/splicing hubs located either concentrically to the initiation hubs or in nuclear speckles proximal to actively transcribed genes. Finally, the high number of long RNA transcripts produced during elongation helps dissolve these transcriptional condensates [42,103,111,150].

An important functional role served by phase separation-mediated transcription of specific genes is rapid adaptation to extracellular stimuli for cell survival. Indeed, the transcriptional condensates observed in vivo are often short-lived, reflecting the highly dynamic nature of the cell’s responses to various environmental signals. For instance, the transcriptional coactivator YAP forms condensates after redistributing into the nucleus upon hyperosmotic stress and reorganizes the genome into clusters of accessible chromatin regions. Such YAP condensates in turn enrich TFs, such as TEAD1, for downstream transcription of YAP target genes that regulate cell proliferation and survival (Figure 4C) [100]. Intranuclear condensates of the closely associated transcriptional coactivator TAZ, which differs from YAP in its ability to phase separate, can also compartmentalize transcriptional machineries, such as TEAD4, BRD4, MED1 and CDK9, to promote TAZ-specific gene expression implicated in growth, development and tumorigenesis, as well as harness the molecular selectivity afforded by LLPS to shield itself against upstream regulators [101]. Moreover, as the nucleo-cytoplasmic shuttling of YAP and TAZ is regulated mechanically, they can act as intranuclear mechano-effectors in conjunction with MLL4, which also promotes transcriptional condensate formation. Interestingly, in Kabuki syndrome, the loss of function of MLL4 disrupts the counter-balancing of Polycomb group (PcG) compartments needed for the proper maintenance of nuclear architecture, leading to increased mechanical stress, reduced nuclear YAP/TAZ levels and, hence, reduced condensate formation [105]. In the case of cellular heat stress, the intracellular heat-shock transcription factor 1 (HSF1) initiates a rapid response involving genome-wide transcriptional reprogramming (such as increased expression of genes encoding heat-shock proteins and chaperones) by forming phase condensates, which can be dissolved by the chaperone protein HSP70 when the cell recovers [107]. When the cell is under proteotoxic stress, HSF1 can also accumulate in nuclear stress bodies via phase separation, which can also be dissolved by HSP70 to increase transcriptional activities and ensure cell survival; those persistent bodies formed during prolonged stress, however, prime the cell for apoptosis [151]. Collectively, these diverse examples demonstrate that phase separation can organize genomic elements in a high-precision manner to serve as transcriptional hubs that activate specific genes in response to diverse biochemical/biophysical cues.

Lastly, apart from transcriptional condensates, DNA damage response (DDR), which is critical for maintaining genomic integrity and stability, can also be regulated by phase separation via DNA repair foci where large amounts of double-strand break (DSB) repair proteins interact at DNA damage sites [95,152,153]. A molecular marker of early DDR is the phosphorylation of histone variant γH2AX mediated by the ATM protein for downstream recruitment of early DDR factors (such as the sensor complex MRN and the DDR adaptor protein MDC1) to facilitate DNA repair [154]. In line with the earlier observation that DSBs in heterochromatic regions are actively relocated to outside the compartment for homologous repair [155], it has recently been found that RAD52 condensates coupled with various nuclear filaments can drive nucleoplasmic flow generation and DNA repair center formation. These RAD52 droplets can undergo fusion, move to the nuclear periphery and dissolve upon completion of repair, all of which are characteristics of LLPS [93]. Another example is the poly-(ADP-ribose) (PAR)-induced DNA repair hub, where PAR polymerase 1 (PARP1) binds to DSB sites to initiate the deposition of PAR, to which FET family proteins nucleate and drive LLPS of the repair hubs during early DDR (Figure 4D). In particular, FUS is involved in PAR-induced DNA repair hubs by undergoing phase separation to recruit key downstream DDR factors, such as 53BP1, KU80, NBS1 and SFPQ, and organize nano-foci of the phosphorylated histone variant γH2AX into higher-order clusters [12,94]. FET family protein condensates formed during early DDR at PAR-seeded repair hubs exclude 53BP1 but remain accessible for MDC1, which is responsible for phosphorylation signal propagation [12]. Given the fact that FUS is required for the relocation of 53BP1 to DNA damage sites and the accumulation of downstream DDR effectors [94], the post-modification state of DDR factors provides an additional layer of control for modulating DNA repair hubs, where the dissolution of PAR-seeded hubs by phosphorylation allows for the recruitment and accumulation of 53BP1 and other genome “caretakers” via ubiquitination [12]. LLPS of 53BP1 promoted by the synthesis of damage-induced long non-coding RNAs (dilncRNAs) can also drive DDR signaling upon DSB via the recruitment of Pol II pre-initiation complex (PIC), MED1 and CDK9 into the 53BP1 condensates [95]. Thus, LLPS enables the cell to achieve precise spatio-temporal control over a series of DDR events. Furthermore, LLPS of 53BP1 at DSBs can organize damaged chromatin and repair factors into larger repair hubs and shield the damaged sites from extensive nucleolytic processing. The formation of these repair hubs also promotes global p53 activation by incorporating p53 into the condensates, pointing to 53BP1′s role in coordinating DNA lesions with global p53-dependent gene activation and cell fate decision in response to DNA damage [96].

## 5. Perspectives and Outlook

Since its initial demonstrations in biological systems more than a decade ago [7,23], phase separation has been found to play pervasive roles in organizing and regulating diverse chromatin-based molecular processes across a wide range of length scales (from the nucleosome level to higher-order chromatin domains), packing states (both heterochromatin and euchromatin) and intranuclear functions (such as transcription, splicing, DNA damage repair, chromatin loop stabilization and telomere maintenance). The unique physico-chemical properties of these phase condensates are harnessed by the cell to accomplish a wide range of chromatin-based regulatory functions in a spatially and temporally controlled manner. In addition to demonstrating the critical importance of intranuclear compartmentalization, these findings also add a new dimension to our existing understanding of the mechanistic modes and features that govern the hierarchical organization of the eukaryotic genome, such as polymer–polymer interactions, local chromatin motions and intranuclear architectural elements [112]. These insights are made possible through the interplay between conceptual advancements in the physics and chemistry of phase separation, comparative investigations across biological systems and the application of quantitative imaging techniques for the characterization of these phenomena with enhanced spatio-temporal resolutions and sensitivity.

Despite that, the quantitative parameters reported in the majority of the previous studies were primarily limited to the size, density, lifetime/recovery half-time, diffusion coefficient and in vitro concentration range/phase diagram associated with these phase condensates (Table 2). While these are certainly critical parameters that characterize a particular type of condensate, many other equally important and revealing physico-chemical properties, such as morphological features (e.g., aspect ratio), surface tension, viscosity, fusion kinetics and critical concentration for phase separation to occur in vivo were often not measured or specifically reported. This deficiency hence calls for more comprehensive and vigorous in vivo quantifications of chromatin-based phase condensates in future studies. In fact, measurements of some of these parameters have already been undertaken in a few of the recent studies; e.g., the aspect ratio [18,45,97], surface tension [95] and viscosity [18,95] of the condensate droplet, as well as the in vivo critical concentration for LLPS [103]. In addition, two recent studies have demonstrated the use of differential diffusion properties between the condensate and its surrounding cellular environment, as well as across the condensate boundary, as a quantitative criterion for validating phase separation in vivo [102,156]. Moreover, in addition to the fluorescence-based imaging methods conventionally used in most phase separation studies (Table 1), other complementary imaging modalities, such as atomic force microscopy (AFM), electron microscopy (EM) and optical tweezers (OTs), can potentially be used in conjunction with fluorescence imaging to probe specific aspects of chromatin-based condensates that otherwise cannot be easily accessed or measured accurately. To that end, EM has been used to quantify the size of FET protein aggregates induced by PAR chains or the degree of nucleosome array compaction by MeCP2 and its mutants with superior spatial resolution [12,88], OTs have been employed to quantitatively probe the dynamics of FUS droplets fusion in vitro [18] and AFM has enabled the direct visualization of DNA–cohesin holocomplex clusters with unprecedented morphological details [52]. These complementary approaches, combined with the potential usage of novel fluorescent probes (such as the recently developed fluorophores AggFluor capable of quantifying a wide range of local viscosity changes with uniform sensitivity [157]), can constitute a systematic, multi-parametric characterization to substantially enhance our confidence when validating phase separation as the mechanism at work in a specific biological system. In addition to imaging only the protein components, novel strategies for imaging and tracking RNAs (such as those based on fluorescent RNAs [158]) or DNA loci (such as those based on CRISPR/Cas labeling [159]) in living cells can also be simultaneously employed to reveal the in situ interactions between the different types of molecular players involved in various intranuclear condensates.

Moreover, despite the seeming “omnipresence” and “omnipotence” of phase separation, it is also important to exercise caution in not over-interpreting findings that could otherwise be attributed to alternative mechanisms. In particular, merely exhibiting phenomenological characteristics of phase condensates may not automatically mean that phase separation is indeed at work until definitive evidence is obtained. For example, it has been shown that herpes simplex virus replication compartments, while possessing many macroscopic properties of liquid-like condensates, are in fact mediated by a transient DNA-binding mechanism distinct from LLPS [156]. Another recent study has found that the formation of condensate-like TF droplets does not enhance transcription activation for a variety of tested synthetic TFs, hence demonstrating that phase separation is not the default multivalent interaction-based mechanism which the cell uses to regulate biological functions, such as transcription [160].

Another key deficiency in many of the previous studies of phase separation has been the inadequate effort in linking quantitative characterizations with the functional consequences of phase separation. For example, there has been evidence showing that enzymatic reaction rates can be significantly increased within condensates as compared to the surrounding milieu [161]. This example, albeit demonstrated using an in vitro model system, highlights that phase separation-mediated sequestration of biomolecules can enhance substrate-specific reactivity beyond that predicted by the law of mass action, which could in turn lead to far-reaching functional consequences. However, given the complexity of the biochemical pathways and interactions involved, correlating phase separation to its in vivo functional outcomes is challenging and often not performed in a sufficiently quantitative manner. A representative effort in this direction is the recent demonstration that the transcriptional activation of an endogenous oncogene requires a narrow optimal working window of IDR–IDR interactions; artificially inducing phase separation by tuning these interactions beyond the optimum will in fact lead to repressed transcription [102]. The importance of this finding is further underscored in light of the fact that many of the previous phase separation studies were performed at concentrations or expression levels far above the endogenous or physiologically relevant ranges (Table 2). Such potential caveats caution against the temptation to over-attribute the significance of phase separation and call for rigorous quantitative interrogations with close-to-native cellular environments and abundance levels when ascribing phase separation as the underlying mechanism, as has already been eloquently argued by others [162].

Finally, the complexity of chromatin-based condensates involving a variety of distinct biomolecular species, as opposed to purely protein-based condensates, also raises concerns as to whether LLPS is still a sufficiently accurate mechanistic model for describing these systems, or if certain modifications are needed to account for the size and structural features of chromatin, as well as the distinct types of interactions involved. For example, the finding that Pol II clusters adopt a variety of shapes in zebrafish is consistent with a model in which regulatory chromatin provides surfaces for liquid condensation at concentrations too low for LLPS to occur and points to an alternative surface condensation mechanism distinct from canonical LLPS [163]. In other cases where chromatin bridging is necessary to initiate condensate formation, PPPS is perhaps more suitable as an alternative mechanism [46], although definitive experimental evidence for PPPS in actual biological systems remains very limited to date. Moreover, the fact that some of these condensates formed via alternative mechanisms can also exhibit liquid-like macroscopic properties [52] poses a challenge to our current notion of the defining characteristics associated with LLPS. As such, there is a strong need for a comprehensive set of quantitative, universally applicable, yet mechanism-specific criteria that can be applied to ascertain the exact physico-chemical process at work in a specific intranuclear system.

Needless to say, the list of chromatin-based condensates surveyed here is certainly not exhaustive, and new discoveries are constantly emerging. Growing evidence also demonstrates that dysregulation of phase separation-mediated mechanisms could lead to various ailments, such as neurodegenerative diseases and cancers [164,165,166]. As such, another future challenge for the field is to go beyond cultured cell systems and probe phase-separation mediated chromatin organization and dynamics in more physiologically relevant contexts, such as developing embryos, live organoids or disease models [167], in order to solidly place phase separation as a versatile regulatory paradigm underlying diverse intranuclear processes in vivo.

## Figures and Tables

**Table 2:** Various phase separation-mediated chromatin structures and processes, the molecular players involved in them, as well as their quantitative characterizations using different imaging methods.

Chromatin-Based Structure	Molecular Species Involved	Mechanistic Role(s) of Phase Separation	Imaging Methods Used	Quantitative Parameters In Vivo		In Vitro Validation /Concentration Range for LLPS	Refs.
				Spatial	Temporal		
High-order chromatin domains	Chromatin	Nucleosome arrays can phase separate under physiological conditions; BRD4 induces LLPS of acetylated chromatin	Live-cell imaging, FRAP, IF	-	-	Yes 50–750 nM (nucleosome)	[41]
	Histone H1	H1 phase separation facilitated by ATP partitions large segments of DNA or polynucleosomes	Live-cell imaging, FRET, FCS, FRAP, IF	-	-	Yes 0.1–0.3 µM	[40]
Constitutive heterochromatin	HP1a/HP1α	LLPS of HP1a/HP1α drives formation of heterochromatin domains	FRAP, LLSM, RICS	-	Diffusion coeff. *D* ~ 1.09 μm^2^ s^−1^	Yes 0.05–1 mg/mL	[84]
		Phosphorylation of HP1α NTD promotes its LLPS by forming higher-order oligomers	Live-cell imaging, FRAP	-	FRAP half-time: τ_1/2_ ~75 s *	Yes 100–200 µM (phosphorylated)	[85,86]
	HP1β	Multivalent interactions between CDs in HP1β complexes with nucleosomes drive LLPS of heterochromatin	Live-cell imaging, FRAP, IF	Size: ~0.5–1 µm *	FRAP half-time: τ_1/2_ ~50–75 s *	Yes 0.8–50 µM (CD)	[86]
	MeCP2	MeCP2 condensates selectively partition HP1α and enhances the separation between heterochromatin and euchromatin	Live-cell imaging, FRAP, IF	Volume: ~1–5 µm^3^ *	FRAP half-time: τ_1/2_ ~10 s *	Yes 2–10 µM	[87]
		MeCP2 competes with histone H1 to form mutually exclusive chromatin condensates	FRAP, IF, EM	Size: ~0.1–0.3 µm *	-	Yes 1.25–10 µM	[88]
	53BP1	53BP1 undergoes LLPS with HP1α to maintain heterochromatin and prevent DNA damage and genomic instability	Live-cell imaging, FRAP, IF	Area (median): 1.243 µm^2^	FRAP half-time: τ_1/2_ ~10–20 s *	Yes ~10 µM	[89]
Facultative heterochromatin	CBX2/PRC1	CBX2 drives LLPS of PRC1; CBX2-PRC1 condensates compact chromatin by concentrating DNA and nucleosomes via direct binding	Live-cell imaging, FRAP, IF	Area: 0.1–0.2 µm^2^ *	FRAP half-time: τ_1/2_ ~35 s *	Yes 0.8–12.5 µM	[90,91]
Chromatin loops	CTCF	CTCF-mediated chromatin loops act as a topological framework for the formation of phase-separated transcriptional condensates at SEs mediated by Pol II	FISH, FRAP, PALM, STORM	-	Lifetimes: ~10 s (transient) >100 s (stable) FRAP half-time: τ_1/2_ ~20 s * (Pol II clusters)	No	[92]
	Cohesin	Cohesin induces phase separation of DNA–cohesin–homocomplex clusters	AFM, FRAP	Size: 1.14 µm (In vitro )	FRAP half-time: τ_1/2_ ~126 s	Yes 1–1000 nM	[52]
DNA damage repair (DDR) hubs	RAD52	Rad52 condensates coupled with nuclear microtubule filaments drive nucleoplasmic flow and DNA repair center formation	Live-cell imaging, FLIP	Area (mean): 0.1–1.2 µm^2^ *	FLIP half-time: τ_1/2_ ~5 s *	Yes 5–20 µM	[93]
	FET-family TFs (FUS/EWS/ TAF15)	FET family TFs form DDR hubs through LLPS on PAR-seeded DNA damage sites in early DDR response and exclude 53BP1	Live-cell imaging, EM, IF, FRAP, OT, SIM	Size: 0.5–2.5 µm * Aspect ratio: ~1	FRAP half-time: τ_1/2_ ~0.2–1 s	Yes 0.1–500 µM	[12,18]
				Other parameters: viscosity: 10–100 mPa·s			
		FUS drives LLPS of DDR hubs to recruit downstream DDR factors and reorganize γH2AX nano-foci in an FUS-dependent manner	Live-cell imaging, IF, SIM	-	Recruitment time: ~40 s	No	[94]
	53BP1	53BP1 nucleates at DNA damage sites and undergoes LLPS to organize damaged chromatin into larger repair compartments and shield it from nucleolytic processing	Live-cell imaging, FRAP, IF, STORM	Size: 0.6–2.8 µm *	FRAP half-time: τ_1/2_ ~2–20 s *	Yes	[95,96]
				Other parameters: viscosity: 2.5 Pa·s; surface tension: γ ~0.5 μN m^−1^			
Transcription-related hubs	MED1/BRD4	MED1 and BRD4 form phase condensates to concentrate transcriptional machineries at SE-regulated genes to activate their transcription, promoted by short RNAs and low RNA levels via positive feedback loops	Live-cell imaging, FISH, FRAP, IF, PALM	Size: 0.2–1.3 µm * Aspect ratio: ~1.1 (In vitro) *	FRAP half-time: τ_1/2_ ~4 s Diffusion coeff.: *D* = 0.14–0.37 μm^2^ s^−1^ Cluster lifetime: ~3–25 s	Yes 0.2–20 µM	[42,97]
	OCT4	OCT4 can phase separate with MED1 or be incorporated into MED1 condensates	Live-cell imaging, FISH, FRAP, IF, PALM	Size: ~0.3 µm *	-	Yes 10–40 µM	[98]
Transcription-related hubs	Pol II/MED1	Pol II and MED1 form clusters of different sizes and lifetimes; large and stable clusters exhibit phase condensate properties and associate with chromatin at SEs in a transcription-dependent manner	Live-cell imaging, FRAP, PALM, LLSM	Cluster size: ~0.1 µm (small) >0.3 µm (large)	Cluster lifetime: ~12 s (transient) >100 s (stable) FRAP half-time: τ_1/2_ ~10 s Sub-diffusitivity: α ~ 0.4	No	[99]
	YAP	YAP redistributes into the nucleus upon hyper-osmotic stress and forms a phase condensate to reorganize chromatin and enrich TFs for transcription of YAP target genes	Live-cell imaging, FRAP, IF, PALM	Size: 0.2–1.6 µm *	FRAP half-time: τ_1/2_ ~1 s *	Yes 40 µM	[100]
	TAZ	TAZ condensates compartmentalize transcription machineries to promote TAZ-specific gene expression and shield themselves against upstream regulators	Live-cell imaging, FRAP, IF, SIM	Size: 0.3–1.2 µm *	FRAP half-time: τ_1/2_ ~0.5–6 s Diffusion coeff.: *D* = 0.11 μm^2^ s^−1^	Yes 10–80 µM	[101]
	FET-family TFs (FUS/EWS/ TAF15)	FET family TFs form condensates at SEs via both homotypic and heterotypic interactions; EWS/FLI1 form transactivation hubs via LLPS to target GGAA microsatellites at SE loci for oncogene activation/expression; TAF15 condensates nucleated by nascent Pol II CTD form transcriptional initiation hubs to activate transcription but exclude phosphorylated Pol II CTD	Live-cell imaging, FCS, FISH, FRAP, IF, LLSM, SPT	Size: 0.2–2 µm *	Recovery time: 7–10 s Residence time in cluster: 5–20 s Diffusion coeff.: *D* ~2 μm^2^ s^−1^ (nucleolus) or ~0.8 μm^2^ s^−1^ (nucleoplasm)	No	[102,103,104]
				Other parameters: critical concentration for LLPS: ~8 µM (cytoplasm) or ~2.6 µM (nucleus) (TAF15); 1–2 µM (cytoplasm and nucleus) (EWS and FUS)			
	MLL4	MLL4 promotes transcriptional condensate formation, which recruits various TFs that regulate nuclear mechanics and chromatin compaction by balancing PcG condensates	Live-cell imaging, IF, STORM	Area: 0.0062–0.013 µm^2^	Cluster lifetime: ~119 s	Yes 1–10 µM	[105]
	KLF4	KLF4 bridges DNA and initiates LLPS via tight and weak binding in an IDR-independent manner	Live-cell imaging, FRAP	Size: 0.5–3 µm *	FRAP half-time: τ_1/2_ ~10–15 s *	Yes 1.5–10 µM	[106]
Transcription-related hubs	HSF1	LLPS of HSF1 promotes chromatin binding and recruitment of transcription apparatus on HSOP gene loci to activate transcription upon heat stress	Live-cell imaging, FISH, FRAP, IF, STORM, SPT	Size: ~0.3 µm	FRAP half-time: τ_1/2_ ~10 s *	Yes 0.125–5 µM	[107]
	YY1	YY1 mediates LLPS to recruit coactivators and promote formation of enhancer clusters to activate FOXM1 gene expression	Live-cell imaging, FISH FRAP, IF	Area: 3–7 µm^2^	FRAP half-time: τ_1/2_ ~3 s *	Yes 2–10 µM	[108]
Co-transcriptional/ splicing hubs	Cyclin T1	Cyclin T1 condensate promotes phosphorylation and recruitment of Pol II CTD, which transitions from transcription initiation condensate to transcription elongation/RNA splicing condensates consisting of SRSF2 or cyclin T1	Live-cell imaging, FISH, IF, LLSM, SPT	Size: 0.5–3 µm *	-	Yes 0.4–6 mg/mL	[109,110]
	SRSF2		Live-cell imaging, FISH, IF, LLSM	Size: ~0.2 µm *	FRAP half-time: τ_1/2_ ~1 s *	Yes 2.5–10 µM	[111]
ALT telomere-associated PML nuclear body (APB)	SUMO–SIM	APB condensates driven by SUMO–SIM LLPS promote telomere clustering in ALT DDR factors are recruited to APB condensates via DDR signaling for telomere elongation induced by DNA damage in ALT	Live-cell imaging, FISH, FRAP, IF	Size: 0.3–1 µm * aspect ratio: ~1 *	FRAP half-time: τ_1/2_ ~35–44 s	No	[45]

*Abbreviations*: AFM: atomic force microscopy; FCS: fluorescence correlation spectroscopy; FISH: fluorescence in situ hybridization; FLIP: fluorescence loss in photobleaching; FRAP: fluorescence recovery after photobleaching; FRET: Förster resonance energy transfer; IF: immunofluorescence; LLSM: lattice light-sheet microscopy; OT: optical tweezers; PALM: photoactivated localization microscopy; RICS: raster image correlation spectroscopy; SIM: structured illumination microscopy; SPT: single-particle tracking; STORM: stochastic optical reconstruction microscopy. * Estimated values from figures in the respective works.
